# Virtual Management of Patients With Cancer During the COVID-19 Pandemic: Web-Based Questionnaire Study

**DOI:** 10.2196/19691

**Published:** 2020-06-24

**Authors:** Emad Tashkandi, Ahmed Zeeneldin, Amal AlAbdulwahab, Omima Elemam, Shereef Elsamany, Wasil Jastaniah, Shaker Abdullah, Mohammad Alfayez, Abdul Rahman Jazieh, Humaid O Al-Shamsi

**Affiliations:** 1 Department of Medical Oncology Oncology Center King Abdullah Medical City Makkah Saudi Arabia; 2 College of Medicine Umm AlQura University Makkah Saudi Arabia; 3 National Cancer Institute Cairo University Cairo Egypt; 4 Oncology Center King Abdullah Medical City Makkah Saudi Arabia; 5 Department of Medical Oncology Oncology Centre Mansoura University Mansoura University Egypt; 6 Princess Noorah Oncology Center King Abdul-Aziz Medical City Ministry of National Guard – Health Affairs Jeddah Saudi Arabia; 7 King Saudi Bin Abdulaziz University for Health Sciences King Abdullah International Medical Research Center Riyadh Saudi Arabia; 8 Department of Oncology Alzahra Hospital Dubai United Arab Emirates; 9 Emirates Oncology Society Dubai United Arab Emirates; 10 Department of Medicine University of Sharjah Sharjah United Arab Emirates

**Keywords:** teleoncology, telemedicine, eHealth, cancer, COVID-19, public health

## Abstract

**Background:**

During the coronavirus disease (COVID-19) pandemic, patients with cancer in rural settings and distant geographical areas will be affected the most by curfews. Virtual management (telemedicine) has been shown to reduce health costs and improve access to care.

**Objective:**

The aim of this survey is to understand oncologists’ awareness of and views on virtual management, challenges, and preferences, as well as their priorities regarding the prescribing of anticancer treatments during the COVID-19 pandemic.

**Methods:**

We created a self-administrated electronic survey about the virtual management of patients with cancer during the COVID-19 pandemic. We evaluated its clinical sensibility and pilot tested the instrument. We surveyed practicing oncologists in Gulf and Arab countries using snowball sampling via emails and social media networks. Reminders were sent 1 and 2 weeks later using SurveyMonkey.

**Results:**

We received 222 responses from validated oncologists from April 2-22, 2020. An awareness of virtual clinics, virtual multidisciplinary teams, and virtual prescriptions was reported by 182 (82%), 175 (79%), and 166 (75%) respondents, respectively. Reported challenges associated with virtual management were the lack of physical exam (n=134, 60%), patients’ awareness and access (n=131, 59%), the lack of physical attendance of patients (n=93, 42%), information technology (IT) support (n=82, 37%), and the safety of virtual management (n=78, 35%). Overall, 111 (50%) and 107 (48%) oncologists did not prefer the virtual prescription of chemotherapy and novel immunotherapy, respectively. However, 188 (85%), 165 (74%), and 127 (57%) oncologists preferred the virtual prescription of hormonal therapy, bone modifying agents, and targeted therapy, respectively. In total, 184 (83%), 183 (83%), and 176 (80%) oncologists preferred to continue neoadjuvant, adjuvant, and perioperative treatments, respectively. Overall, 118 (53%) respondents preferred to continue first-line palliative treatment, in contrast to 68 (30%) and 47 (21%) respondents indicating a preference to interrupt second- and third-line palliative treatment, respectively. For administration of virtual prescriptions, all respondents preferred the oral route and 118 (53%) preferred the subcutaneous route. In contrast, 193 (87%) did not prefer the intravenous route for virtual prescriptions. Overall, 102 (46%) oncologists responded that they would “definitely” prefer to manage patients with cancer virtually.

**Conclusions:**

Oncologists have a high level of awareness of virtual management. Although their survey responses indicated that second- and third-line palliative treatments should be interrupted, they stated that neoadjuvant, adjuvant, perioperative, and first-line palliative treatments should continue. Our results confirm that oncologists’ views on the priority of anticancer treatments are consistent with the evolving literature during the COVID-19 pandemic. Challenges to virtual management should be addressed to improve the care of patients with cancer.

## Introduction

In December 2019, a cluster of patients with severe pneumonia were identified in Wuhan, China, and a novel coronavirus disease (COVID-19) was discovered [1]. This disease can range from asymptomatic infection to severe respiratory distress syndrome and death. The World Health Organization has declared the COVID-19 outbreak a pandemic. As of April 25, 2020, a total of 2,719,897 cases and 187,705 confirmed deaths have been reported across more than 200 countries [2].

This highly contagious virus is characterized by rapid human-to-human transmission [3], and risk factors for mortality include older age and comorbidities [4]. Patients with cancer are susceptible to COVID-19 infections because of the immunosuppressive effect of cancer treatments like chemotherapy or surgery [5], and hence have a poorer prognosis.

Teleoncology is the application of telemedicine to oncology. It has the potential to enhance access to and improve the quality of clinical cancer care [6]. Patients from rural and distant geographical areas will be most affected by curfews. Virtual management (telemedicine) has been shown to reduce health costs and improve access to care. There are examples of successful technology applications for survivorship care, palliative care, symptoms management, and supervision of satellite anticancer infusion suites [7-9]. An updated conceptual framework of telemedicine during the COVID-19 pandemic has been defined and could be applied at larger scale to improve national public health responses [10]. Further reduction of patients’ exposure to infection could be achieved by replacing certain clinic visits with virtual clinics (via videoconference or telephone) to minimize hospital visits. This allows oncologists to defer routine follow-ups, assess patients who can continue certain anticancer treatments (such as chemotherapy, immunotherapy, targeted therapy, or hormonal therapy), and continue cancer care virtually.

Multidisciplinary tumor boards ensure the selection of high priority curative cases and improve outcomes without delays or interruptions of cancer care. This could be continued virtually, depending on availability and the capacity of the health care system.

Virtual prescription and delivery of drugs is an alternative way to manage patients with cancer, especially when delivering drugs to their home via courier services or to health facilities near their home to avoid interruption of treatments, provided that this service is logistically feasible and available.

Oncologists need to weigh the risks and benefits of anticancer treatments during the pandemic. Caring for patients with cancer during this period is challenging. Jeopardizing safety by exposing patients to infection when they leave their home to visit oncology clinics and receive anticancer treatments may lead to greater risks of potential adverse events.

There is a limited number of studies to guide oncologists on how to manage patients with cancer during a pandemic. In this survey, we aim to report the views of oncologists on virtual management (awareness, challenges, and preferences) and their priorities when prescribing anticancer treatments during the COVID-19 pandemic. This could help oncologists conduct future controlled studies or trials, and guide health systems on areas of improvement in supportive infrastructure.

## Methods

### Study Design

This study presents the findings of a web-based questionnaire submitted to licensed oncologists in the Gulf and Arab regions.

### Study Population

We included study subjects who met the following criteria: licensed practicing oncologists in the Gulf or Arab regions who are treating adult or pediatric patients, and involved in the care of patients with cancer using anticancer treatments (eg, chemotherapy, novel immunotherapy, targeted therapy, hormonal therapy, and bone modifying agents). Exclusion criteria were nononcologists and trainees.

### Study Procedures

We used a nonprobability snowball sampling [11] design. To identify our target population, we contacted oncologists who are members of established national oncology associations and societies in the region to distribute and participate in the survey. If this was not applicable, we contacted 1 to 2 regional oncologists per area to distribute and participate in the survey. We used email and WhatsApp, the most popular social media network in the region, to reach oncologists.

### Development of the Instrument

We generated our survey instrument using rigorous survey development and testing methods [12]. Items were selected based on a literature review, as well as email and telephone correspondence. In total, 5 experts in the field of oncology and hematology from 3 countries extensively discussed the topic and reviewed items until no further questions were missed. Items were nominated and then ranked by expert oncologists to reach a consensus on the selected items. Further review was done by methodology and content experts to eliminate redundant items using binary responses (exclude and include). We aimed to have a survey that is simple, succinct, and easy to understand. During the construction of the survey, we grouped the items into domains we wanted to explore and then refined the questions [13]. The self-administered survey (Multimedia Appendix 1) consisted of 20 items that focused on 4 domains: characteristics of oncologists; COVID-19 pandemic measures; virtual management and oncologists’ views on virtual management; and the priority of prescribing anticancer treatments.

The structured response formats used in this survey included binary (yes/no), nominal, and ordinal responses. Other options were also allowed, including “Undetermined,” “Other,” and any other comments with free text to capture unanticipated responses. Respondents received electronic links accompanied with concise instructions, a cover letter is stating the background, the objectives of the survey, the target population, and a request to participate voluntarily (that stated their answers will be kept anonymously using SurveyMonkey).

### Testing of the Instrument

During pretesting and pilot testing, questions were reviewed by 3 colleagues specializing in oncology to check the consistency and appropriateness of the questions designed by investigators [14,15], and were then reviewed by nonexpert colleagues to assess the dynamics, flow, and accessibility. In total, 5 oncology members carried out pilot testing of the instrument with minor modifications. We also conducted a clinical sensibility assessment to evaluate the comprehensiveness, clarity, and face validity of our instrument on a scale of 1 to 5. For this assessment, we invited 5 colleagues with methodologic and oncology expertise. The results of the clinical sensibility assessment, which used the mean scores indicated on a 5-point scale, suggested that the instrument had face validity (4.4), content validity (4.2), clarity (4.6), and discriminability (4.3).

### Administration of the Instrument

After the approval of the King Abdullah Medical City Institutional Research Board, we sent the questionnaires electronically to licensed oncologists in the region who treat adult or pediatric patients. Oncologist types included medical oncologists, malignant hematologists, pediatric oncologists, clinical oncologists, and hemato-oncologists.

### Study Duration and Timeline

On April 2, 2020, we sent participants an embedded link to the web-based survey on SurveyMonkey (along with an electronic cover letter with instructions to complete the survey) via emails, text messages, and social media such as Facebook, Twitter, and WhatsApp. Primary investigators contacted the oncology members of national associations and societies in the region to participate and create a broad distribution network. Regional oncologists distributed the survey link to their regional members and network; there were no incentives provided. We sent reminders 1 and 2 weeks later, and we closed the survey on April 22.

### Statistical Analysis

Descriptive statistics were used to summarize data, and synthesize and report the views of oncologists. Description of the data also included proportions, frequencies, means, and standard deviation for continuous variables when appropriate.

## Results

We received 222 completed surveys from 10 different countries in the region (Table 1). Overall, 71% (n=157) of respondents were males. Respondents have been in oncology practice for a median of 10 years. The top respondent specialty was medical oncology (n=97, 44%). Saudi Arabia is the country of current practice for 47% (n=105) of respondents. The remaining respondents practice in Arab countries. In total, 74% (n=163) practice in the public health sector and 97% (n=215) practice in urban locations.

The 222 respondents were asked whether there are any diagnosed COVID-19 cases in their country, city, hospital, and department (Table 2). In total, 97% (n=215), 97% (n=215), and 77% (n=172) of respondents indicated that there were cases in the country, city, and hospital in which they practice, respectively. In total, 18% (n=41) of respondents reported COVID-19 cases among their own patients.

**Figure 1:**
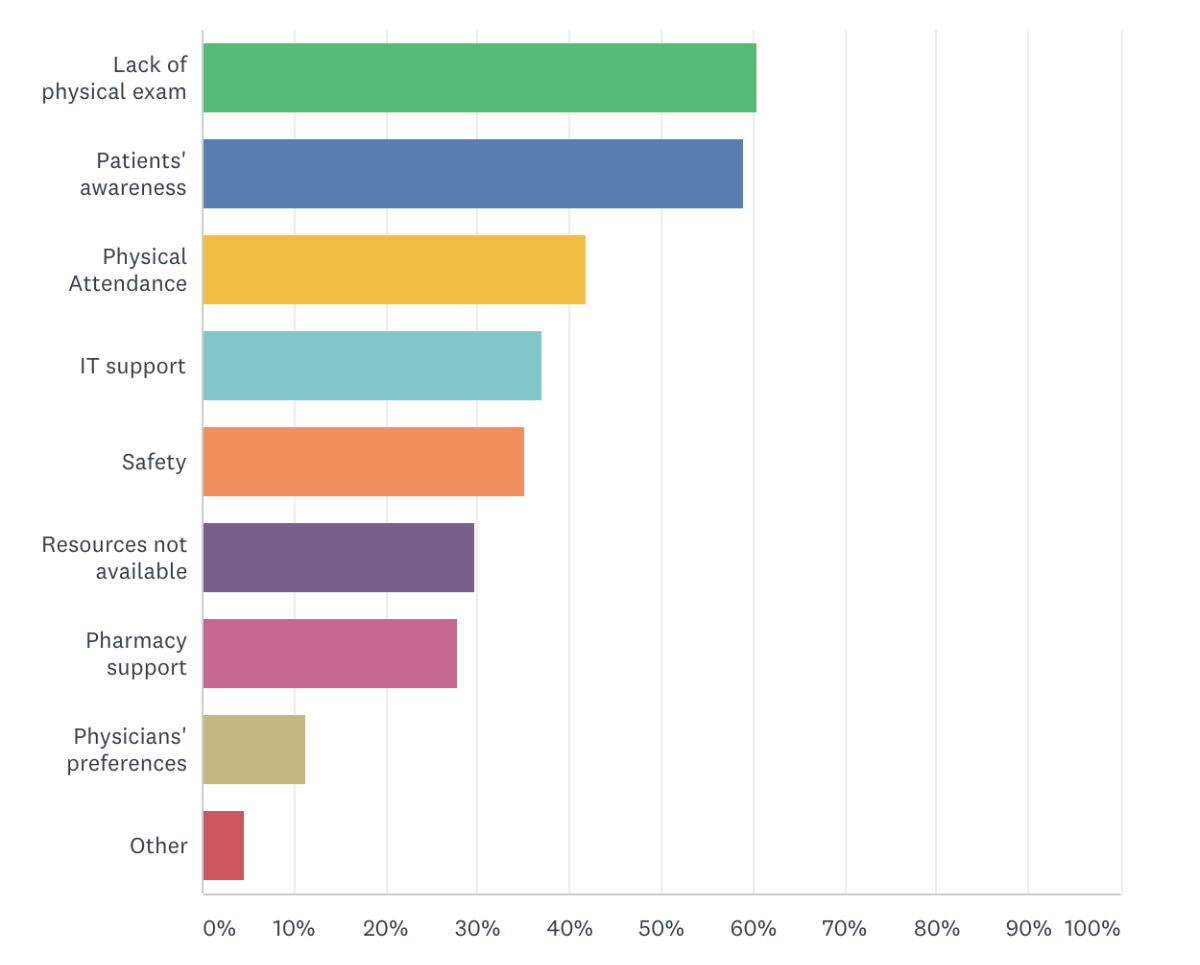
Respondents’ reported challenges regarding virtual management. Respondents were requested to select more than one response, if applicable. IT: information technology.

Overall, out of 222 respondents, 91% (n=210) regularly attend multidisciplinary tumor boards with a monthly multidisciplinary tumor board number of 4 or more reported by 56% (n=125). In total, 82% (n=182), 79% (n=175), and 75% (n=166) of respondents were aware of virtual clinics, virtual multidisciplinary tumor boards, and virtual prescription, respectively (Table 3). Additionally, 59% (n=131), 64% (n=142), and 64% (n=143) of respondents have been personally involved in a virtual clinic, virtual multidisciplinary tumor board, and virtual prescription and delivery of drugs, respectively. Challenges faced by respondents regarding virtual management were the lack of physical examination (n=134, 60%), patients’ awareness and access (n=131, 59%), the lack of physical attendance of patients (n=93, 42%), information technology (IT) support (n=82, 37%), and safety (n=78, 35%). Overall, 5% (n=10) had other comments such as the lack of a direct doctor-patient encounter, medicolegal aspects, psychological support, and privacy (Figure 1).

**Table 1:** Characteristics of respondents (N=222).

Characteristics		Respondents
**Gender, n (%)**		
	Male	157 (71)
	Female	65 (29)
Years in oncology practice, median		10
**Specialty, n (%)**		
	Medical oncologist	97 (44)
	Hematologist	31 (14)
	Oncologist and hematologist	29 (13)
	Clinical oncologist	28 (13)
	Pediatric oncologist	27 (12)
	Other	10 (5)
**Practicing country, n (%)**		
	Saudi Arabia	105 (47)
	United Arab Emirates	38 (17)
	Egypt	18 (8)
	Tunisia	13 (6)
	Kuwait	10 (5)
	Lebanon	8 (4)
	Bahrain	7 (3)
	Oman	5 (2)
	Jordan	4 (2)
	Other	12 (5)
**Practice setting, n (%)**		
	Public health care	163 (73)
	Private health care	33 (15)
	Both public and private	26 (12)
**Practice location, n (%)**		
	Urban	215 (97)
	Rural	7 (3)

**Table 2:** Respondents’ responses to questions about confirmed coronavirus disease cases.

Questions	Responses		
	Yes, n (%)	No, n (%)	Unknown, n (%)
Are there coronavirus disease cases in the country in which you are practicing?	215 (97)	6 (2.5)	1 (0.5)
Are there coronavirus disease cases in the city in which you are practicing?	215 (97)	6 (2.5)	1 (0.5)
Are there coronavirus disease cases in the hospital in which you are practicing?	172 (77)	41 (18)	9 (4)
Are there coronavirus disease cases in your department?	54 (24)	152 (68)	16 (7)
Are there coronavirus disease cases among your own patients?	41 (18)	165 (74)	16 (7)

**Table 3:** Respondents’ reported awareness about virtual management.

Virtual management type	Awareness	Personally involved
Virtual clinic	182 (82)	131 (59)
Virtual tumor board	175 (79)	142 (64)
Virtual prescription and delivery of drugs	166 (75)	143 (64)

**Figure 2:**
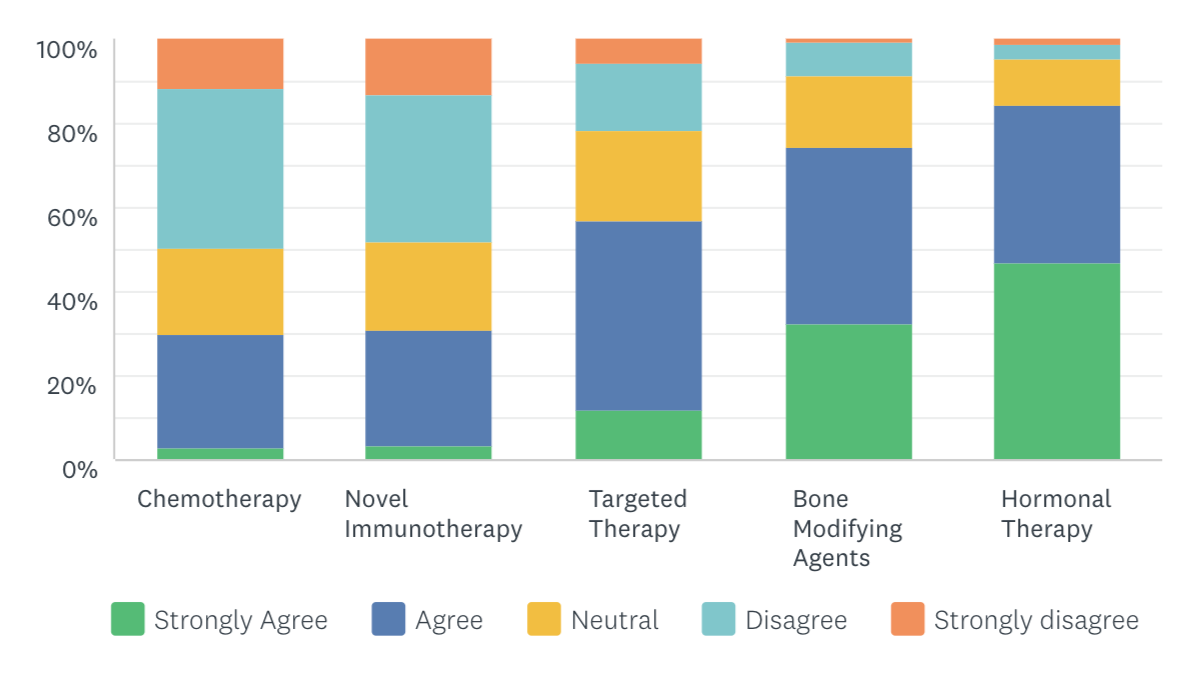
Respondents’ reported anti-cancer treatments that can be prescribed virtually.

When asked about the priority of prescribing anticancer treatments during the COVID-19 pandemic, 50% (n=111) and 48% (n=107) of the 222 surveyed oncologists indicated they did not prefer the virtual prescription of chemotherapy and novel immunotherapy, respectively (Figure 2). However, 85% (n=188), 74% (n=165), and 57% (n=127) of oncologists preferred the virtual prescription of hormonal therapy, bone modifying agents, and targeted therapy, respectively (Table 4).

**Figure 3:**
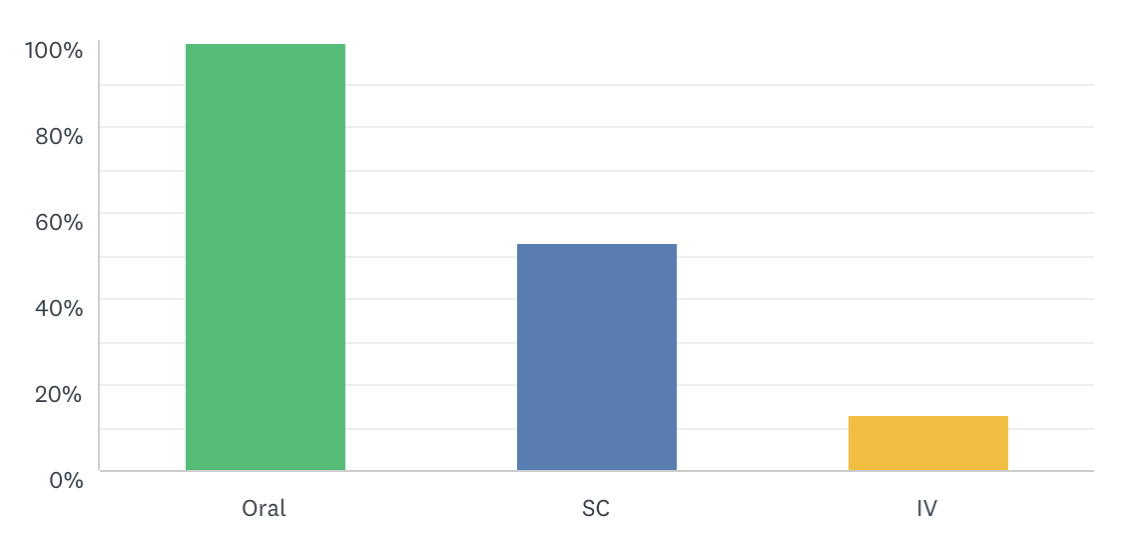
Respondents’ reported anti-cancer treatments that can be prescribed virtually, by route. IV: intravenous. SC: subcutaneous.

When prescribing treatments virtually, all 222 respondents preferred treatments that are administered by the oral route and 53% (n=118) preferred the subcutaneous route. In contrast, 87% (n=193) of oncologists did not prefer the intravenous route for virtual prescriptions (Figure 3).

**Figure 4:**
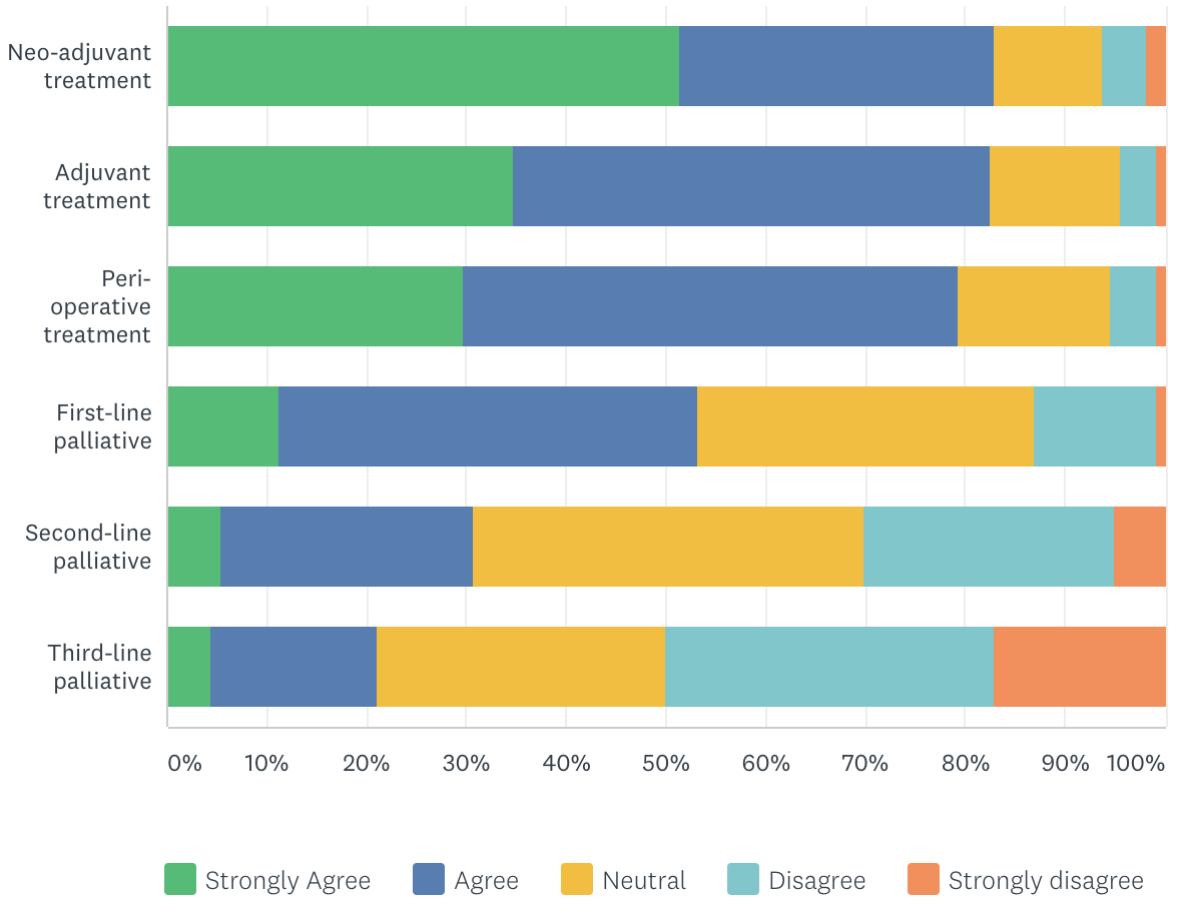
Respondents’ responses regarding which anti-cancer treatments should not be interrupted.

Of 222 respondents, more than 80% of oncologists preferred to continue neoadjuvant (n=184, 83%), adjuvant (n=183, 83%), and perioperative (n=176, 80%) treatments (Table 5). In addition, 53% (n=118) preferred to continue first-line palliative treatment. In contrast, 30% (n=68) and 21% (n=47) preferred to interrupt second- and third-line palliative treatment, respectively (Figure 4).

**Figure 5:**
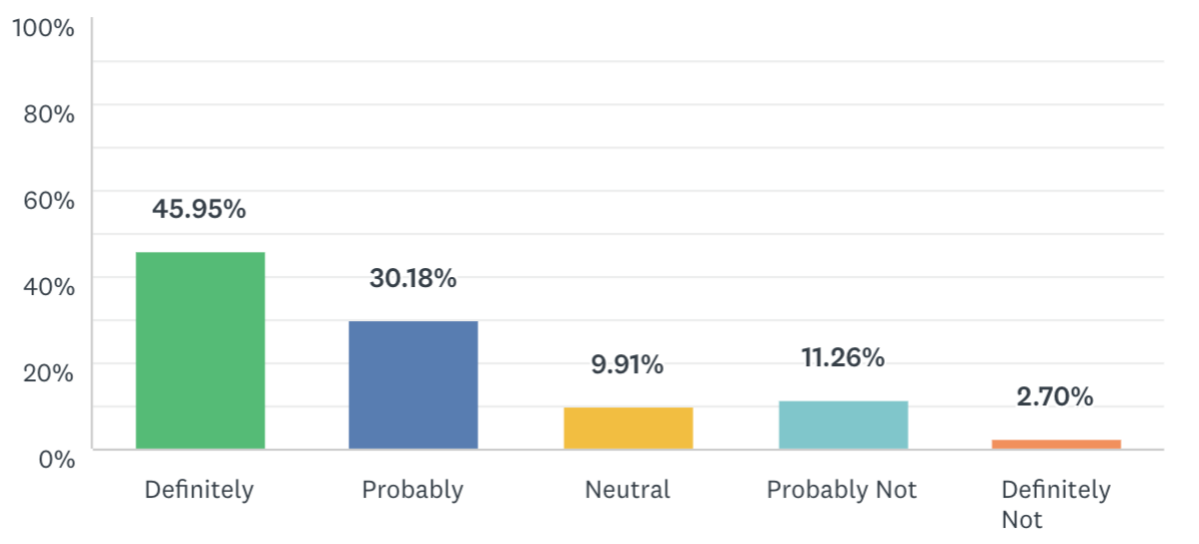
Respondents’ responses when asked if they prefer to manage cancer patients virtually.

When the 222 oncologists were asked if they prefer to manage cases virtually, 46% (n=102) responded “Definitely,” 30% (n=67) responded “Probably,” 10% (n=22) answered “Neutral,” 11% (n=25) said “Probably not,” and 3% (n=6) said “Definitely not” (Figure 5). In total, 40% (n=87) of respondents reported that patients were satisfied with virtual management, while 18% (n=40) indicated patients were not satisfied, and 43% (n=95) answered “I don’t know.” Overall, 36% (n=80) of respondents indicated they are likely to continue virtual management after the pandemic, while 51% (n=112) said they will not, and 14% (n=30) answered “I don’t know.”

**Table 4:** Respondents’ reported anticancer treatments that can be prescribed virtually.

Anticancer treatments	Strongly Agree, n (%)	Agree, n (%)	Neutral, n (%)	Disagree, n (%)	Strongly Disagree, n (%)
Chemotherapy	7 (3)	60 (27)	45 (20)	84 (38)	26 (12)
Novel immunotherapy	8 (4)	61 (27)	46 (21)	78 (35)	29 (13)
Targeted therapy	27 (12)	100 (45)	47 (21)	36 (16)	12 (5)
Hormonal therapy	104 (47)	84 (38)	24 (110)	8 (4)	2 (10)
Bone modifying agents	72 (32)	93 (42)	38 (17)	18 (8)	1 (0.4)

**Table 5:** Respondents’ reported anticancer treatments that should not be interrupted.

Treatments	Strongly Agree, n (%)	Agree, n (%)	Neutral, n (%)	Disagree, n (%)	Strongly Disagree, n (%)
Neoadjuvant	114 (51)	70 (32)	24 (11)	10 (5)	4 (2)
Adjuvant	77 (35)	106 (48)	29 (13)	8 (4)	2 (1)
Perioperative	66 (30)	110 (50)	34 (15)	10 (5)	2 (1)
First-line palliative	25 (11)	93 (42)	75 (34)	27 (12)	2 (1)
Second-line palliative	12 (5)	56 (25)	87 (39)	56 (25)	11 (5)
Third-line palliative	10 (5)	37 (17)	64 (29)	73(33)	38 (17)

## Discussion

### Overview

During the COVID-19 curfew, oncologists need to weigh the risks and benefits of anticancer treatments. Patients from distant geographical areas are affected the most. Virtual management (telemedicine) has been shown to reduce health costs and improve access to care. Examples of successful technology applications include symptoms management and supervision of satellite anticancer infusion suites [7]. To our knowledge, oncologists’ views about virtual management during the COVID-19 pandemic have not been described previously.

Our work demonstrates that oncologists have a high level of awareness of virtual clinics, virtual multidisciplinary teams, and virtual prescriptions (82%, 79%, and 75%, respectively). However, despite this high level of awareness, oncologists’ actual involvement was significantly lower, as shown in Table 3. We presume these differences are related to the major challenges of virtual management faced by respondents, as shown in Figure 1. Challenges mentioned included a lack of physical examination (60%), patient’s awareness and access (59%), a lack of physical attendance of patients (42%), IT support (37%), and the safety of virtual management (35%).

Nonetheless, we found that 46% of the surveyed oncologists responded that they “definitely” prefer to manage some cases virtually. However, only 36.0% will continue virtual management after the pandemic; we cannot explain why the proportion is low, although challenges and preferences with virtual management might be potential reasons.

No studies have previously described oncologists’ views about the priority of anticancer treatments during the COVID-19 pandemic. The results of our survey demonstrated that more than 80% of oncologists preferred to continue neoadjuvant, adjuvant, and perioperative treatments. Additionally, 53% reported their preference to continue first-line palliative treatment, in contrast to 20% and 30% that preferred to interrupt second- and third-line palliative treatment, respectively. These results are consistent with Hanna et al [16], where the proposed resources utilization, allocation, and prioritization of anticancer treatments indicated a high priority for curative treatments versus a low priority for palliative treatments. Of note, this model has not been tested in clinical studies. Other studies have shown that delaying adjuvant treatments was associated with inferior survival in colon cancer [17] and breast cancer [18].

Changing the drug administration route from intravenous to oral without compromising outcomes has been reported in the literature [19]. In our study, we found that 50% and 48% of oncologists did not prefer the virtual prescription of chemotherapy and novel immunotherapy, respectively; the majority are parenteral drugs. However, 85%, 74%, and 57% of oncologists preferred the virtual prescription of hormonal therapy, bone modifying agents, and targeted therapy, respectively; the majority are oral drugs. All respondents preferred the oral route, in keeping with Hofheinz et al [19], and 53% preferred the subcutaneous route for virtual prescription. In contrast, 87% of oncologists did not prefer the intravenous route for virtual prescription.

This study has several strengths. First, we described the views of oncologists on virtual management and the priority of anticancer treatments during the COVID-19 pandemic, which has not been reported previously. Second, we used a rigorous methodology for our instrument development, validation, and administration, as no appropriate instrument previously existed. Third, we used virtual snowball sampling to identify experts in the field of oncology in the region, as there are no lists or other obvious sources for locating all practicing oncologists who are members of societies or nonmembers. It is difficult to estimate the total size of the sample.

The limitations of our study include that the number of participants in the study was relatively small, and they were mostly from Saudi Arabia. One inherent weakness of this study is its restricted participation to the Arab world, which limits the inferences that can be drawn from the data. Another important limitation is that there were differences in respondent specialties, which included medical oncology, hematology, pediatric oncology; half of the study group specialized in medical oncology. However, we tried to control for this by inviting more respondents to participate. Future research could be done with more specialties and the involvement of oncologists from different geographic regions.

Our study adds to the previous knowledge that oncologists have a high level of awareness about virtual management, although the doctors have lower actual involvement in virtual clinics, virtual multidisciplinary tumor boards, and virtual prescriptions. Our results confirm that oncologists’ views on the priority of anticancer treatments are consistent with the evolving literature during the COVID-19 pandemic.

Virtual management could be implemented as an evolving method to manage a selected group of patients with cancer who live in remote locations. Mclean et al [20] showed no differences in outcomes between telehealth and usual care. This would reduce the risk associated with hospital visits and of infection transmisson. Examples of successful implementation include survivorship care, palliative care, symptoms management, and supervision of satellite anticancer infusion suites [7-9]. Similarly, this could be implemented for patients with routine follow-ups who have a low risk of relapse, as well as for patients receiving oral treatment, hormonal treatment, and bone modifying agents.

Challenges to virtual management should be addressed to improve the care of patients with cancer and to enhance oncologists’ actual involvement in virtual management. This can be done by improving patients’ awareness and access, improving IT support, assessing the safety of virtual management, and finding solutions to the need for physical attendance and physical examination. A number of questions remain unanswered, such as the safety of virtual management. Cancer care is complex and encompasses the need for direct doctor-patient encounters, clinical examination, medicolegal aspects, psychological support, privacy, and adequate infrastructure to support logistics. In addition, virtual management may only be feasible and applicable at some institutions. These are possible avenues for future research and will allow us to understand how these factors translate into the improvement of cancer care.

Taken together, virtual management is an evolving tool for caring for patients with cancer under certain circumstances. If it is implemented in the appropriate venues, it will improve access to care [6] and reduce the health care burden on patients with cancer. Virtual prescription of anticancer treatments during the COVID-19 pandemic has not been addressed before and is worth pursuing in further research.

### Conclusions

In this regional survey, we found that oncologists have a high level of awareness about virtual management, with lower actual involvement with virtual clinics, virtual multidisciplinary tumor boards, and virtual prescriptions. Oncologists indicated that second- and third-line palliative treatments should be interrupted, while neoadjuvant, adjuvant, perioperative, and first-line palliative treatments should continue. Our results confirm that oncologists’ views on the priority of anticancer treatments are consistent with the evolving literature during the COVID-19 pandemic. Challenges to virtual management should be addressed to improve the care of patients with cancer.

## Appendix

Multimedia Appendix 1 Survey questionnaire.

## Abbreviations

COVID-19: coronavirus disease
IT: information technology

## References

[1] Zhu N, Zhang D, Wang W, Li X, Yang B, Song J, Zhao X, Huang B, Shi W, Lu R, Niu P, Zhan F, Ma X, Wang D, Xu W, Wu G, Gao GF, Tan W (2020). A Novel Coronavirus from Patients with Pneumonia in China, 2019. N Engl J Med.

[2] World Health Organization Coronavirus disease 2019.

[3] Zhou F, Yu T, Du R, Fan G, Liu Y, Liu Z, Xiang J, Wang Y, Song B, Gu X, Guan L, Wei Y, Li H, Wu X, Xu J, Tu S, Zhang Y, Chen H, Cao B (2020). Clinical course and risk factors for mortality of adult inpatients with COVID-19 in Wuhan, China: a retrospective cohort study. The Lancet.

[4] Liang W, Guan W, Chen R, Wang W, Li J, Xu K, Li C, Ai Q, Lu W, Liang H, Li S, He J (2020). Cancer patients in SARS-CoV-2 infection: a nationwide analysis in China. Lancet Oncol.

[5] Shankar A, Saini D, Roy S, Mosavi Jarrahi A, Chakraborty A, Bharti SJ, Taghizadeh-Hesary F (2020). Cancer Care Delivery Challenges Amidst Coronavirus Disease–19 (COVID-19) Outbreak: Specific Precautions for Cancer Patients and Cancer Care Providers to Prevent Spread. Asian Pac J Cancer Prev.

[6] Hazin R, Qaddoumi I (2010). Teleoncology: current and future applications for improving cancer care globally. The Lancet Oncology.

[7] Sirintrapun SJ, Lopez AM (2018). Telemedicine in Cancer Care. Am Soc Clin Oncol Educ Book.

[8] Al-Shamsi HO, Alhazzani W, Alhuraiji A, Coomes EA, Chemaly RF, Almuhanna M, Wolff RA, Ibrahim NK, Chua ML, Hotte SJ, Meyers BM, Elfiki T, Curigliano G, Eng C, Grothey A, Xie C (2020). A Practical Approach to the Management of Cancer Patients During the Novel Coronavirus Disease 2019 (COVID-19) Pandemic: An International Collaborative Group. Oncologist.

[9] Khairat S, Meng C, Xu Y, Edson B, Gianforcaro R (2020). Interpreting COVID-19 and Virtual Care Trends: Cohort Study. JMIR Public Health Surveill.

[10] Ohannessian R, Duong TA, Odone A (2020). Global Telemedicine Implementation and Integration Within Health Systems to Fight the COVID-19 Pandemic: A Call to Action. JMIR Public Health Surveill.

[11] Aday LA, Cornelius LJ (2006). Designing and conducting health surveys: a comprehensive guide.

[12] Argüelles Méndez L (2016). From Lisp to FuzzyLisp. Studies in Fuzziness and Soft Computing.

[13] Passmore C, Dobbie AE, Parchman M, Tysinger J (2002). Guidelines for constructing a survey. Fam Med.

[14] Collins D (2003). Pretesting survey instruments: an overview of cognitive methods. Qual Life Res.

[15] Woodward CA (1988). Questionnaire construction and question writing for research in medical education. Med Educ.

[16] Hanna TP, Evans GA, Booth CM (2020). Cancer, COVID-19 and the precautionary principle: prioritizing treatment during a global pandemic. Nat Rev Clin Oncol.

[17] Biagi JJ, Raphael MJ, Mackillop WJ, Kong W, King WD, Booth CM (2011). Association between time to initiation of adjuvant chemotherapy and survival in colorectal cancer: a systematic review and meta-analysis. JAMA.

[18] Raphael MJ, Biagi JJ, Kong W, Mates M, Booth CM, Mackillop WJ (2016). The relationship between time to initiation of adjuvant chemotherapy and survival in breast cancer: a systematic review and meta-analysis. Breast Cancer Res Treat.

[19] Hofheinz R, Wenz F, Post S, Matzdorff A, Laechelt S, Hartmann JT, Müller L, Link H, Moehler M, Kettner E, Fritz E, Hieber U, Lindemann HW, Grunewald M, Kremers S, Constantin C, Hipp M, Hartung G, Gencer D, Kienle P, Burkholder I, Hochhaus A (2012). Chemoradiotherapy with capecitabine versus fluorouracil for locally advanced rectal cancer: a randomised, multicentre, non-inferiority, phase 3 trial. Lancet Oncol.

[20] McLean S, Sheikh A, Cresswell K, Nurmatov U, Mukherjee M, Hemmi A, Pagliari C (2013). The impact of telehealthcare on the quality and safety of care: a systematic overview. PLoS One.

