# Supplementary material for: Virtual Management of Patients With Cancer During the COVID-19 Pandemic: Web-Based Questionnaire Study
Source: J Med Internet Res. 2020 Jun 24;22(6):e19691. doi: 10.2196/19691 (PMC7316128; doi:10.2196/19691)
Supplement: Multimedia Appendix 1 [file jmir_v22i6e19691_app1.docx]

**Appendix 1 survey questionnaire**

The Pandemic of COVID-19 affected the oncologists and their patients. Some of the cancer patients need to continue their treatment without interruptions. Lockdown of the cities and travel restrictions could affect treatment decisions further. Virtual management could be used as an alternative way to help oncologists and patients with regards to treatment decisions.

The goal of this survey to understand the views of oncologists about virtual management (awareness, challenges and preferences) and priority of anti-cancer treatment that can be prescribed virtually during the COVID-19 pandemic.

This survey is intended for Oncologists (medical oncologists, malignant hematologists and pediatric oncologists)

You are invited to participate in this survey (5 minutes to complete).

You are under no obligation to participate. The information collected will not be shared or linked to any identifying information you provided and will be kept anonymous

Thank you for supporting this project.

Do you agree to participate in this study?

Yes

No

Gender? Select one

Male

Female

Are you actively involved in direct cancer care including prescription of chemotherapy?

Yes

No

Have you completed your training and you are fully licensed to practice and work independently (Residents and fellows are not eligible to participate)?

Yes

No

What best describes you? Select one

Medical oncologist

Oncologist and hematologist

Clinical oncologist (provide both Chemotherapy and Radiation therapy)

Hematologist

Gynecologist

Pediatric oncologist

Internal medicine physician practicing in oncology

Other

Which country are you mainly practicing in? (if you are practicing in more than one, then choose the one with most time practice being held at): Select one

Saudi Arabia

UAE

Egypt

Jordan

Lebanon

Oman

Kuwait

Bahrain

Algeria

Tunis

Other

How many years since you completed your training?

……

Do you work in: Select one

Public health care setting

Private health care setting

Both public and private

Are you practicing in a rural or urban location? Rural defined as: two hours away from a tertiary cancer center and population of less than 50,000 Select one

Urban

Rural

On average month, I participate in the multidisciplinary tumor board for:

0 (We do not have access to multidisciplinary tumor board) Select one

1

2

3

4+

Yes, No, Unknown:

Do you have COVID cases diagnosed in the country you are practicing?

Do you have COVID cases diagnosed in the city?

Do you have COVID cases diagnosed in the Hospital?

Do you have COVID cases diagnosed in your specialty?

Do you have COVID cases diagnosed in your patients?

Are you aware of the following? Yes or No

- Virtual clinic: communicate with patients via audio visual applications that include but not limited to “AVAYA, Zoom, Skype Google duo, Tango, Hangouts, Allapp etc”.
- Virtual tumor board: communicate with tumor board members via audio visual applications that include but not limited to “AVAYA, Zoom, Skype, Google duo, Tango, Hangouts, Allapp etc.
- Electronic prescriptions and or delivery of drugs to patients (for example after virtual clinic you prescribe the drug and will be delivered to the patient’s home)

Have you been actually involved in any of the following, you can select more than one?

- Virtual clinic
- Virtual tumor board
- Electronic prescriptions and or delivery of drugs to patients

Do you prefer virtual management for some of your cancer patients?

- Definitely
- Probably
- Neutral
- Probably Not
- Definitely Not

What challenges you face, you can select more than one?

- Resources not available
- IT support
- Pharmacy support
- Safety of virtual management
- Physicians preferences
- Patients awareness & access
- You prefer to see the patient physically

During COVID-19 Pandemic, which of the following treatment you could prescribe virtually, you can select more than one?

- Chemotherapy (Strongly Agree, Agree, Neutral, Disagree, Strongly disagree)
- Novel Immunotherapy (Strongly Agree, Agree, Neutral, Disagree, Strongly disagree)
- Targeted therapy (Strongly Agree, Agree, Neutral, Disagree, Strongly disagree)
- Hormonal therapy (Strongly Agree, Agree, Neutral, Disagree, Strongly disagree)
- Bone modifying agents (Strongly Agree, Agree, Neutral, Disagree, Strongly disagree)

During COVID-19 Pandemic, which of the following routes you could prescribe virtually, you can select more than one?

- Oral
- IV
- SC

During COVID-19 Pandemic, which of the following treatment, DO NOT WANT TO INTERRUPT, you can select more than one?

- Neoadjuvant treatment (Strongly Agree, Agree, Neutral, Disagree, Strongly disagree)
- Adjuvant treatment (Strongly Agree, Agree, Neutral, Disagree, Strongly disagree)
- Perioperative treatment (Strongly Agree, Agree, Neutral, Disagree, Strongly disagree)
- 1st line palliative therapy (Strongly Agree, Agree, Neutral, Disagree, Strongly disagree)
- 2nd line palliative therapy (Strongly Agree, Agree, Neutral, Disagree, Strongly disagree)
- 3rd line palliative therapy (Strongly Agree, Agree, Neutral, Disagree, Strongly disagree)

Final comments?

End of survey, Thank you so much
